# Metabolic Plasticity of Tumor Cell Mitochondria

**DOI:** 10.3389/fonc.2018.00333

**Published:** 2018-08-24

**Authors:** Giuseppe Cannino, Francesco Ciscato, Ionica Masgras, Carlos Sánchez-Martín, Andrea Rasola

**Affiliations:** Department of Biomedical Sciences, University of Padova, Padova, Italy

**Keywords:** mitochondria, tumor metabolism, signal transduction, oxidative phosphorylation, neoplastic growth, oncometabolites, redox homeostasis, calcium

## Abstract

Mitochondria are dynamic organelles that exchange a multiplicity of signals with other cell compartments, in order to finely adjust key biological routines to the fluctuating metabolic needs of the cell. During neoplastic transformation, cells must provide an adequate supply of the anabolic building blocks required to meet a relentless proliferation pressure. This can occur in conditions of inconstant blood perfusion leading to variations in oxygen and nutrient levels. Mitochondria afford the bioenergetic plasticity that allows tumor cells to adapt and thrive in this ever changing and often unfavorable environment. Here we analyse how mitochondria orchestrate the profound metabolic rewiring required for neoplastic growth.

## Introduction

Mitochondria are metabolic hubs that harbor enzymes responsible for several biochemical circuitries, including tricarboxylic acid (TCA) cycle, oxidative phosphorylation (OXPHOS), fatty acid oxidation (FAO), biosynthesis of amino acids, lipids and nucleotides and maintenance of homeostatic levels of Ca^2+^ and of reducing equivalent carriers. These bioenergetic, biosynthetic and signaling functions render mitochondria capable of rapidly sensing and integrating stress signals, in order to coordinate biochemical pathways required for the appropriate responses of the cell to environmental changes (1).

Mitochondria gained center stage in molecular oncology when Otto Warburg observed that tumor cells can ferment glucose to lactate even in the presence of oxygen, proposing that a failure in mitochondrial respiration was the cause of this metabolic trait, called aerobic glycolysis, and that this was in turn required for neoplastic growth (2, 3). Decades after this groundbreaking observation, we know that aerobic glycolysis is part of a wider metabolic rewiring that characterizes neoplastic growth. During this process, environmental conditions can rapidly fluctuate, following local changes in oxygen, pH or nutrient gradients, and can become extremely harsh for the transformed cell, which must become capable of tackling sudden shortages in blood supply or exposure to anti-neoplastic treatments.

**Figure 1:**
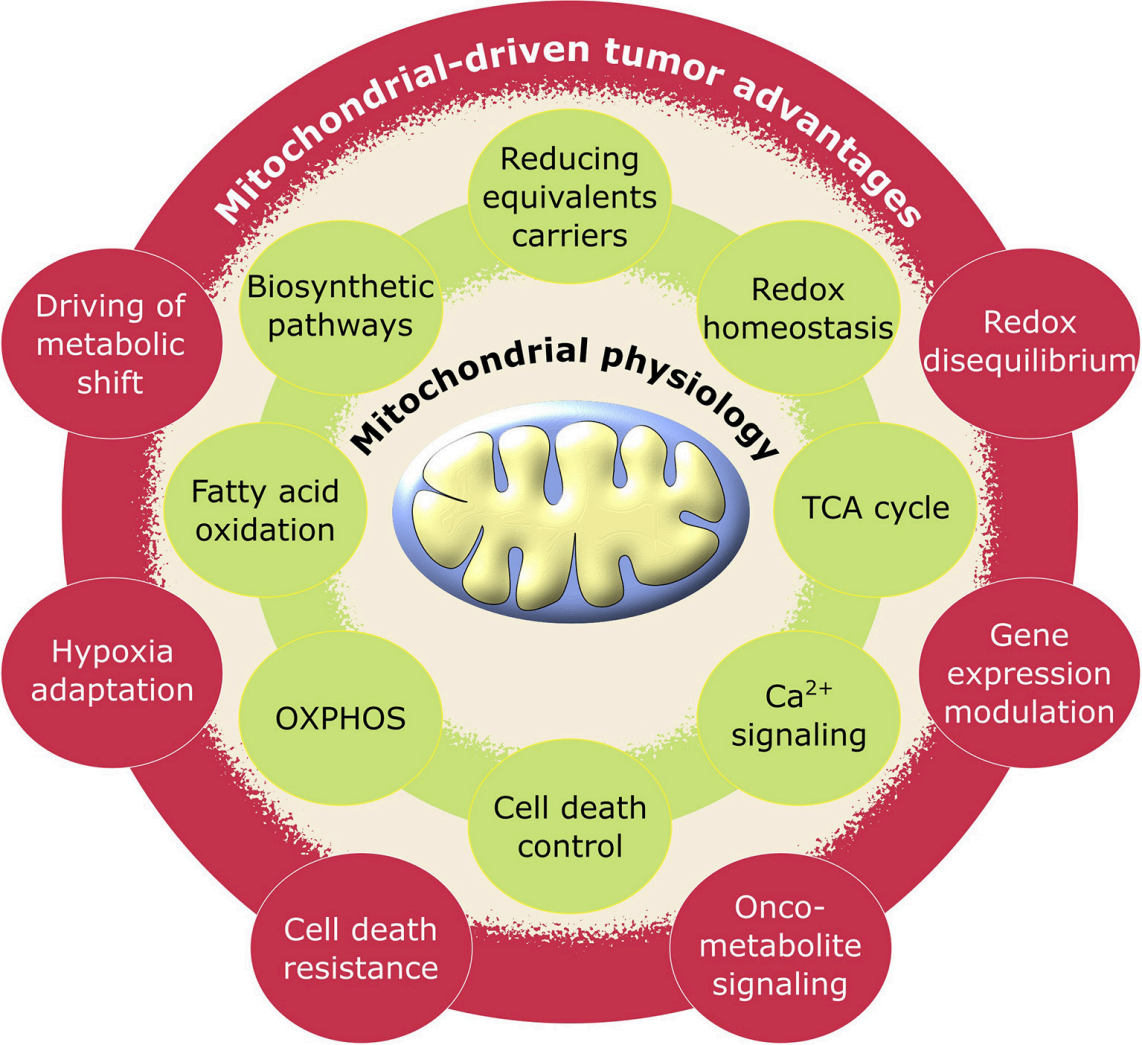
Schematic representation of pro-tumoral biological processes regulated by mitochondria. Mitochondrial physiology (green) acquires advantageous alterations in cancer (red) adjusting its metabolic activity to support the requirements for neoplastic cell growth and proliferation.

**Figure 2:**
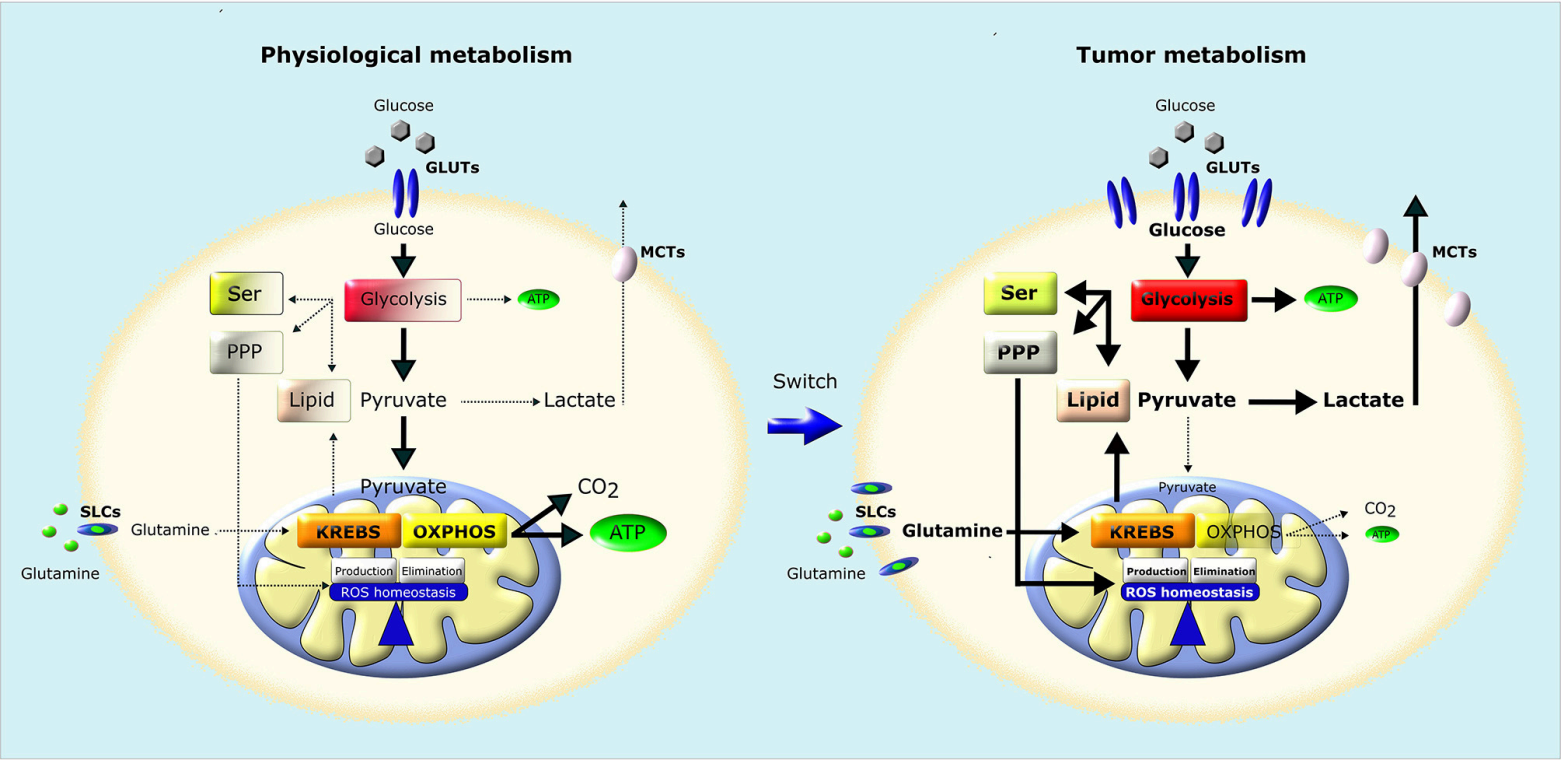
Metabolic remodeling of cancer cells. In normal cells **(left)**, a large fraction of glucose is metabolized to pyruvate that is almost completely oxidized to CO_2_ through TCA (Krebs) cycle and OXPHOS in mitochondria, producing a large amount of ATP. Pyruvate is metabolized to lactate only in conditions of limiting O_2_. Instead, most cancer cells **(right)** convert most glucose to lactate regardless of O_2_ availability (Warburg effect). The increased glucose utilization through glycolysis, associated to an increase in glutamine utilization, generates metabolic intermediates used for the synthesis of nucleic acids through pentose phosphate pathway (PPP), serine biosynthesis pathway (SER) and lipid biosynthesis, providing the building blocks for the anabolic needs of cancer cells. In addition, neoplastic cells undergo an increase in ROS generation, and therefore increase their antioxidant defenses to avoid oxidative damage and maintain ROS homeostasis. GLUTs, Glucose Transporters; MCTs, Lactate Transporters; SLCs, Solute Carriers.

Unlike Warburg's proposal, tumor cell mitochondria not only retain their functionality, but are also instrumental for integrating a variety of signals and adjusting the metabolic activity of the cell to such a demanding and stressful situation (4) (Figure 1). OXPHOS activity is down-regulated, but not abolished, in many tumor cell types. Therefore, malignant cells start producing a large portion of their ATP through glycolysis rather than OXPHOS. Enhanced glucose utilization also increases the metabolic flux through pentose phosphate pathway (PPP) (5), which provides anabolic building blocks for nucleotide synthesis and NADPH for anti-oxidant defenses, whereas glycolytic intermediates are used for the biosynthesis of amino acids (4, 6–8). These metabolic changes down-regulate the TCA cycle, both because induction of PPP and of anabolic pathways that branch from glycolysis limit pyruvate availability, and because a low OXPHOS activity inhibits the formation of NAD^+^ and FAD required for TCA cycle dehydrogenases. Thus, mitochondria must activate anaplerotic mechanisms in order to feed the TCA cycle, as its activity is required for fatty acid (FA) and amino acid biosynthesis and for the homeostatic maintenance of reducing equivalent carriers (6, 9–14); this is mainly achieved by increasing the pace of glutamine utilization (9, 15) (Figure 2).

Several recent lines of evidence suggest that mitochondria indeed play a key promoter role in tumor growth and progression (16). All along this process, mitochondrial biogenesis and quality control are often upregulated, and mitochondria can even retain a high level of OXPHOS in some tumor cell types. Rare human neoplasms with defective respiration caused by mutations in mitochondrial genome, such as oncocytomas (17, 18), are relatively benign, and mitochondrial DNA depletion impairs tumorigenicity in several tumor cell models (19). Altogether, these observations imply the existence of a negative selection for a loss of mitochondrial function in neoplastic transformation (20).

Mitochondrial bioenergetics is largely under the control of extra-mitochondrial biochemical pathways, whose activity is often altered by oncogenic mutations (21). Moreover, some metabolic alterations that directly originate from mitochondria are oncogenic *per se* (22). In certain tumor settings, mitochondria can act as neoplastic drivers by generating high levels of oncometabolites, *i.e*., metabolites that are able to change the genomic and epigenomic landscape of the cell, hence prompting the tumorigenic process (23, 24). Thus, the crosstalk between mitochondria and rest of the cell can amplify the metabolic drift of tumor cells away from their non-transformed counterparts during neoplastic progression.

In the present review, we analyse how the metabolic plasticity of tumor cell mitochondria contributes to the neoplastic process. However, any general consideration must be confronted with the real scenario of a tumor mass, where a myriad of factors influence metabolism. These include the tissue of origin of the neoplastic cell, its mutational and epigenomic profile and the local environmental conditions, which can dictate confined changes in the bioenergetic features of the cells, prompting metabolic heterogeneity even in different portions of the same tumor, or in different moments of its growth (25).

## Oncogenic signalling pathways and mitochondria

**Figure 3:**
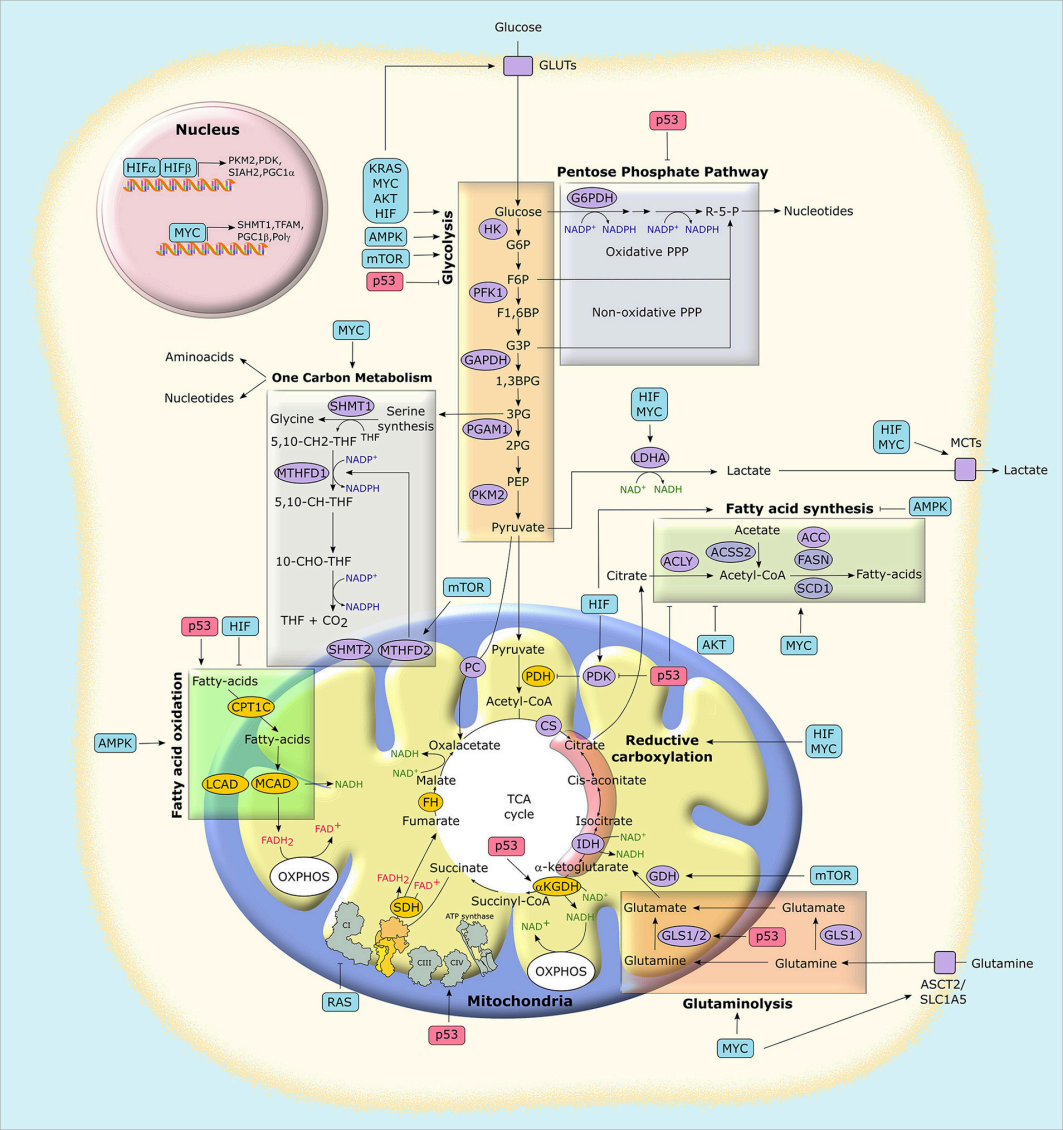
Mitochondria at the crossroad of metabolic networks and signaling cascades. Several proteins with pro-neoplastic activity (in light blue boxes) alter the expression and/or the activity of metabolic enzymes and transporters, thus rewiring the metabolic status of cancer cells. Similarly, the possible loss of the tumor suppressor p53 (in light red boxes) impacts on cancer metabolism at several levels. Enzymes that catalyze metabolic reactions are shown in ovals. Yellow ovals indicate enzymes that are preferentially inhibited in tumors, while purple ovals indicate those that are mostly induced. G6P, glucose-6-phosphate; F6P, fructose-6-phosphate; F1,6BP, fructose-1,6-bisphosphate; G3P, glyceraldehyde-3-phosphate; 1,3BPG, 1,3-bisphosphoglycerate; 3PG, 3-phosphoglycerate; 2PG, 2-phosphoglycerate; PEP, phospho-enolpyruvate; R-5-P, ribose-5-phosphate; MCT, monocarboxylate transporter; GLUT, glucose transporter; PC, pyruvate carrier; ASCT2, alanine, serine, cysteine-preferring transporter 2; 5,10-CH2-THF, 5,10-methylenetetrahydrofolate; 10-CHO-THF, 10-formyl-THF; 5,10-CH-THF, 5,10 methenyl-THF; HIF, hypoxia-inducible factor; HK, hexokinase; PFK1, phosphofructokinase 1; PGAM1, phosphoglycerate mutase 1; PKM2, pyruvate kinase M2 isoform; LDHA, lactate dehydrogenase A; GAPDH, glyceraldehyde-3-phosphate dehydrogenase; PDH, pyruvate dehydrogenase complex; PDK, pyruvate dehydrogenase kinase; αKGDH, α-ketoglutarate dehydrogenase; CS, citrate synthase; SDH, succinate dehydrogenase; FH, fumarate hydratase; IDH, isocitrate dehydrogenase; GLS, glutaminase; ACLY, ATP-citrate synthase; ACSS2, Acyl-coenzyme A synthetase short-chain family member 2; ACC, acetyl-CoA carboxylase; FASN, fatty acid synthase; SCD1, stearoyl-CoA desaturase 1; SHMT, serine hydroxymethyltransferase; MTHFD, methylenetetrahydrofolate dehydrogenase; CPT, carnitine O-palmitoyltransferase; MCAD, medium-chain acetyl-CoA dehydrogenase; LCAD, long-chain acetyl-CoA dehydrogenase; PGC1, peroxisome proliferator-activated receptor gamma, coactivator-1; TFAM, mitochondrial transcription factor A; PPP, pentose phosphate pathway; TCA, tricarboxylic acid; OXPHOS, oxidative phosphorylation.

A complex network of signals moves back and forth between nucleus and mitochondria (Figure 3). This crosstalk constantly keeps under strict nuclear control any mitochondrial function, ensuring its proper harmonization with the metabolic status of the cell. Several major transduction pathways have a strong impact on mitochondrial function, including the transcriptional programs coordinated by HIF1, c-Myc and p53, as well as Ras and mTOR/AMPK signaling (4, 21, 26, 27). Consequently, pro-neoplastic dysregulation of any of these signaling axes strongly affects the mitochondrial metabolic machinery.

### Hypoxia-inducible factors (HIFs) and mitochondrial metabolism

HIFs induce transcription under low oxygen conditions and are active when their two subunits, aryl hydrocarbon receptor nuclear translocator (ARNT, or HIF-1β) and either HIF-1α or HIF-2α, bind hypoxia-responsive elements (HREs) in gene promoters. While ARNT is constitutively expressed, HIF-1α/2α undergo proteasomal degradation triggered by hydroxylation of specific proline residues. The prolyl-hydroxylases (PHDs) targeting HIF-1α/2α are dioxygenases inhibited in hypoxic or anoxic conditions, which leads to stabilization of HIF-1α and/or HIF-2α. HIF stabilization orchestrates a transcriptional program that equips tumor cells to sustain hypoxic stress by affecting several aspects of cancer biology, including angiogenesis, epithelial-to-mesenchymal transition, metastasis, resistance to anticancer therapies as well as metabolic reprogramming (28–30).

HIF-dependent metabolic rewiring embraces induction of glycolysis and FA synthesis together with OXPHOS down-regulation, a key adaptation to low oxygen (31), and has profound effects on mitochondrial activity (Figure 3). One of the glycolytic enzymes induced by hypoxia is hexokinase type II (HK II), the most active hexokinase isoform whose expression is upregulated in many cancer types and contributes to their efficiency in glucose utilization (32, 33). In tumor cells, HK II is mainly anchored to the outer mitochondrial membrane, and its detachment from mitochondria rapidly induces cell death (34–36). Thus, mitochondrial binding of HK II has an important tumorigenic function (37) and displays a protective role for mitochondrial function and cell viability through mechanisms yet poorly defined, but involving autophagy regulation in conditions of glucose paucity (38).

The transcriptional program mastered by HIFs creates a bottleneck in funneling glycolysis toward the TCA cycle by slowing-down the conversion of pyruvate to acetyl-CoA (31). This is achieved both through induction of the M2 isoform of pyruvate kinase (PKM2), which is less active than the M1 counterpart in generating pyruvate from phosphoenolpyruvate, and by eliciting the expression of pyruvate dehydrogenase kinase 1 (PDK1), an inhibitor of the pyruvate dehydrogenase complex (PDC) (39, 40). In addition, HIFs promote lactate dehydrogenase (LDHA) expression, again pushing pyruvate away from the TCA cycle toward its conversion into lactate, using reducing equivalents provided by glycolysis-derived NADH and thus keeping the NAD^+^ levels required for a sustained glycolytic activity (41). The parallel induction of monocarboxylic acid transporters (MCTs) causes lactate extrusion from the cell and contributes to acidification of the surrounding environment. As a combined result of these modulations, OXPHOS activity is down-modulated, and glycolytic intermediates upstream to pyruvate accumulate and can be diverted to anabolic routines (42).

In these conditions, tumor cells must use lipids and amino acids as main metabolic fuels (43), finding glucose-independent sources for acetyl-CoA generation required for *de novo* FA synthesis and for acetylation reactions (see section Post Translational Regulation In Cancer Metabolism). In general, tumor cells increase FA synthesis and the intracellular levels of total FAs for membrane synthesis, lipid signaling or as energy source (when oxidized) (44, 45). HIF signaling increases lipid uptake and the induction of lipid kinases and oxidases, resulting in an overall dysregulation of lipid metabolism in cancer (46). To obtain high levels of acetyl-CoA, mitochondria of cells undergoing hypoxia boost reductive carboxylation of glutamine (47), which generates citrate via the TCA cycle enzymes isocitrate dehydrogenase (IDH) and aconitase. Citrate then moves to cytosol, where it can be cleaved into oxaloacetate and acetyl-CoA by ATP citrate lyase (ACLY), thus starting FA synthesis (Figure 3). HIF1α causes proteasomal degradation of a subunit of the α-ketoglutarate dehydrogenase (αKGDH) complex, a TCA component that is responsible for oxidative glutamine metabolism, by inducing the E3 ubiquitin-ligase SIAH2 (48). Thus, HIF-dependent transcription enhances reductive carboxylation of glutamine by inhibiting its oxidation. In parallel with induction of FA synthesis, HIF signaling down-modulates FAO both directly, by inhibiting the expression of the mitochondrial enzymes medium- and long-chain acetyl-CoA dehydrogenase (MCAD and LCAD) (49) and indirectly, by inducing PHD3, which activates acetyl-CoA carboxylase 2 (ACC2), thus prompting generation of the FAO repressor malonyl-CoA (50).

Mitochondria can also directly regulate HIF stability in a process termed pseudohypoxia that is independent of environmental oxygen levels and further adds flexibility to the metabolic responses of tumor cells (see section Mutations Of Mitochondrial Enzymes In Cancer Metabolism). Furthermore, at least in a model of renal carcinoma, HIF1α can repress the expression of PGC-1α (peroxisome proliferator-activated receptor gamma, coactivator-1α), a central regulator of mitochondrial biogenesis, which in turn stabilizes HIF1α (51). These observations highlight the existence of regulatory loops between mitochondria and the transcriptional program mastered by HIFs (52). Hypoxia also creates a redox stress in mitochondria, as oxygen is the final electron acceptor in OXPHOS and inadequate oxygen levels increase the leakage of electrons out of respiratory complexes, forming reactive oxygen species (ROS). Therefore, HIF signaling is also involved in the maintenance of redox homeostasis, another complex bioenergetic adaptation required for neoplastic progression in which mitochondrial play a central role (see section Redox Homeostasis And Mitochondrial Metabolism In Tumors).

### c-Myc and mitochondrial metabolism

c-Myc is one of the most frequently induced oncogenes in human cancers, where its transcriptional function becomes constitutively activated following deregulation of oncogenic pathways, gene amplification or chromosomal translocation (53). The effect of c-Myc activation is the orchestration of nutrient uptake and cell growth and proliferation, making its dysregulation a key oncogenic driver. These biological routines require a robust anabolic induction, and this is crucially supported by mitochondria. There are several ways by which c-Myc affects mitochondrial metabolism, thus sustaining growth of neoplastic cells in the unfavorable environment they must deal with. The transcriptional program mastered by c-Myc partially overlaps the metabolic effects of HIF-dependent signaling. Indeed, c-Myc upregulates the same set of glycolytic genes that are targeted by HIFs, including GLUT1, LDHA, MCTs, PKM2, and HK II, thus increasing glucose uptake and its utilization both in glycolysis and PPP (Figure 3). As discussed for HIFs, these changes cause a metabolic rewiring toward aerobic glycolysis, lowering in parallel pyruvate availability for the TCA cycle and OXPHOS (54).

At variance from HIFs, however, c-Myc is active under non-hypoxic conditions, and can stimulate mitochondrial biogenesis and respiration. c-Myc activates mitochondrial transcription factor A (TFAM), PGC1β and mitochondrial DNA polymerase gamma, which elicit the expression of hundreds of genes encoding for mitochondrial proteins (55). This could be relevant for the local adaptations of tumor cells to the microenvironmental heterogeneity they find in the tumor mass. It is possible to envision that c-Myc can prompt both glycolysis and OXPHOS in neoplastic cells located in the proximity of blood vessels, where high levels of oxygen are available. Instead, when cells encounter more hypoxic conditions, c-Myc could cooperate with HIFs in increasing glycolysis and attenuating mitochondrial OXPHOS, without inhibiting other mitochondrial metabolic activities (56).

Induction of mitochondrial serine hydroxymethyltransferase (SHMT2) by c-Myc provides an elegant example of this conditional cooperation between c-Myc and HIFs in regulating metabolic circuitries of tumor cell mitochondria. SHMT2 is the major source of the one-carbon unit required for folate metabolism and for the biosynthesis of nucleotides and amino acids (Figure 3). It utilizes serine, obtained from the glycolytic intermediate 3-phosphoglycerate, and tetrahydrofolate (THF) to catalyze the synthesis of glycine and 5,10-methylenetetrahydrofolate (5,10-CH2-THF). In turn, 5,10-CH2-THF can generate formate and the reducing equivalent donor NADPH in a reaction catalyzed by methylenetetrahydrofolate dehydrogenase 2 (MTHFD2). c-Myc-dependent induction of SHMT2 under normoxic conditions has important biosynthetic consequences: formate is released in the cytosol where it is involved in purine synthesis; glycine and 5,10-CH2-THF contribute to nucleotide synthesis, and NADPH is required for reductive biosynthesis of amino acids, deoxyribonucleotides and lipids (54, 55). When a tumor cell faces hypoxia, HIF stabilization further induces SHMT2. This is counterintuitive, as in low-oxygen conditions cells inhibit proliferation, thus reducing demand for anabolic fluxes. Nevertheless, under hypoxia SHMT2 is essential for survival of c-Myc-transformed cells as it protects them from oxidative stress. Indeed, MTHFD2 activity maintains a high NADPH:NADP^+^ ratio, and NADPH is required for regeneration of the antioxidant tripeptide glutathione and hence for protection from ROS damage (57). Notably, SHMT2 is required for survival and proliferation of neoplastic cells in ischemic tumor zones (58).

c-Myc also promotes the glutamine addiction that characterizes several cancer cell types (54, 55) (Figure 3). Glutamine is both a nitrogen and carbon source essential for biomass accumulation, and a substrate used for bioenergetic purposes, and it is avidly consumed by neoplastic cells for proliferation and survival (Figures 2, 3). c-Myc increases the expression of the plasma membrane glutamine transporter, ASCT2/SLC1A5, and of glutaminases (GLS) that convert glutamine to glutamate in cytosol (GLS1) or mitochondria (GLS2) as a first step of its oxidation (15, 59). Glutamate generates α-ketoglutarate (α-KG) either via glutamate dehydrogenase (GDH), in a reaction that releases ammonia, or via several aminotransferases that transfer glutamate nitrogen to α-keto acids, such as pyruvate, for producing other amino acids and α-KG. In turn, α-KG feeds the TCA cycle, which is therefore accelerated by c-Myc via glutaminolysis induction. c-Myc also increases the TCA cycle flux up to four-folds by eliciting the expression of most of its enzymes (55). When the TCA cycle flux is impaired, e.g., in low glucose conditions or by mutations in some of its components, α-KG can act as an anaplerotic substrate that provides carbon units to yield citrate by moving backwards through the TCA cycle through reductive carboxylation (9). In cytosol, citrate starts lipid synthesis. c-Myc induces the string of enzymes responsible for the first steps of lipidogenesis, whose upregulation occurs across most tumors (45). These enzymes include ACLY, which uses citrate to synthesize acetyl-CoA, Acetyl-CoA carboxylase, which generates malonyl-CoA, fatty acid synthase and stearoyl-CoA desaturase (55). Malonyl-CoA inhibits carnitine acyl transferase I (*aka* carnitine palmitoyltransferase I, CPT I), the carrier responsible for the uptake of fatty acids in mitochondria, thus acting in a feedback loop on mitochondrial metabolism to inhibit FAO (60).

Taken together, these observations demonstrate how tumor cell mitochondria can control the homeostatic balance of reducing equivalent donors, OXPHOS activity, lipid synthesis and oxidation in response to c-Myc and/or HIF activation, with crucial implications for chromatin remodeling, tuning of all major anabolic pathways and handling of oxidative insults.

### p53 and mitochondrial metabolism

The transcription factor p53 is a key tumor suppressor activated by a set of stress signals, such as genotoxic damage, oncogene activation, nutrient or oxygen scarcity and loss of cell-to-cell contacts, all of which characterize malignant transformation (61, 62). A functional inactivation of p53 occurs in the majority of tumor types (63). p53 mainly exerts its activity in the nucleus by regulating the expression of genes or microRNAs (miRNAs), even though it can also act in cytosol and mitochondria to inhibit autophagy (64) or to promote cell death (65). p53 activation induces death or senescence when a sustained or intense stress causes irreversible cell damage. Conversely, mild stresses result in p53-dependent adaptive responses, consistent with its role in repairing or avoiding damage. During fluctuations in oxygen or nutrient availability, the effect of p53 is a global promotion of cell catabolism, associated with an inhibition of proliferation and growth (Figure 3). p53 interacts with mTOR (mammalian target of rapamycin) and AMPK (AMP-activated protein kinase), two master regulators of cellular metabolism. The mTORC1 complex is active in the presence of both adequate growth conditions and mitogens and coordinates the anabolic responses of the cell (66), whereas AMPK inhibits mTORC1 and allows cells to adapt to energetic stresses by utilizing glucose and FA for increasing ATP levels (see below) (67). A complex interplay exists between p53 and the mTORC1/AMPK circuitry: p53 both activates AMPK and elicits the expression of several negative regulators of mTORC1, whereas AMPK activates p53 through several means, including its phosphorylation and acetylation (62, 68). In this way, cells can respond in a balanced and flexible way to metabolic variations, different types of stress signals, growth and proliferation inputs (69).

p53 can also exert a more direct action on several metabolic effectors (Figure 3). In general, it dampens the glycolytic rate through a concerted downregulation of glucose transporters (GLUTs) (70) and of the glycolytic enzyme phosphoglycerate mutase (PGAM) (71). p53 also prompts the expression of TIGAR (TP53-induced glycolysis and apoptosis regulator), which indirectly inhibits phosphofructose kinase 1 (PFK1) (72), thus leading to a p53-dependent diversion of glycolytic intermediates into the PPP (62). However, the effect of p53 on glucose metabolism is highly context-dependent, as p53 can also negatively modulate PPP activity (73). In parallel to inhibiting glycolysis, p53 enhances mitochondrial bioenergetic activity in several ways (Figure 3). It promotes mitochondrial quality control by inducing mitophagy to substitute damaged mitochondria, and it increases mitochondrial DNA copy number and mitochondrial mass (68). p53 boosts TCA cycle via induction of the mitochondrial glutaminase GLS2 (74, 75), thus fueling glutamine to glutamate conversion and in turn α-KG generation through the TCA cycle enzyme α-KGDH. Moreover, p53 activates the PDC as it inhibits the negative PDC regulator pyruvate dehydrogenase kinase 2 (76), increasing pyruvate funneling into the TCA cycle through its conversion to acetyl-CoA. p53 also enhances OXPHOS activity by inducing the expression of subunit I of cytochrome c oxidase (COX), the complex IV of the electron transport chain, and of the COX assembly factor SCO2, as well as of apoptosis-inducing factor (AIF), which is required for a proper OXPHOS functioning (68).

In general, p53 lifts lipid utilization inhibiting FA synthesis and increasing mitochondrial FAO in a coordinated manner (68). This results from p53-dependent modulation of glycolysis and PPP, as these routines supply the building blocks (acetyl-CoA and NADPH) needed for lipid synthesis. Furthermore, p53 directly stimulates FAO by inducing the mitochondrial membrane FA transporters carnitine acetyltransferases (CPTs) (77, 78), the transcriptional co-activator for FAO genes lipin 1 (79), and pantothenate kinase 1, which is essential in β-oxidation as it is involved in CoA biosynthesis (80). Moreover, p53 induces malonyl-CoA decarboxylase, which lowers the levels of the CPT allosteric inhibitor malonyl-CoA (81). Thus, p53 opposes the metabolic shift toward the induction of FA synthesis and glycolysis that characterizes many tumor cell types and supports mitochondrial FAO and OXPHOS. Notably, FAO induction further feeds OXPHOS via generation of FADH2 and NADH. Nonetheless, under conditions of extreme stress p53 can have an opposite effect on mitochondria, potently contributing to their dysfunction through repression of the master regulators of mitochondrial biogenesis PGC-1α/PGC-1β, thus leading to cell senescence or death (82).

### The AMPK/mTOR system and mitochondrial metabolism

AMPK acts as a homeostatic device whose purpose is the rapid restoration of energy balance through an orchestrated inhibition of ATP-consuming biosynthetic pathways. It activates following a drop in ATP levels, as it senses the AMP/ATP ratio. Therefore, AMPK induction is promoted by down-regulation of mitochondrial OXPHOS activity; in turn, AMPK induces glucose uptake and glycolysis while inhibiting storage of glucose, and it suppresses FA synthesis by phosphorylating and inhibiting ACCs (83) (Figure 3). ACC2, the isozyme associated to the outer mitochondrial membrane, generates malonyl-CoA that inhibits CPT1 and therefore mitochondrial import of FAs for their oxidation. Hence, ACC2 inhibition by AMPK induces mitochondrial FAO (67). Moreover, AMPK inhibits *de novo* lipid generation via direct phosphorylation of SREBP1 (sterol regulatory element binding protein 1), a master transcriptional regulator of lipid synthesis (84). AMPK also prompts mitophagy through phosphorylation of ULK kinases (1) and mitochondrial fragmentation through phosphorylation of mitochondrial fission factors (85) in order to restrict energy expenditure and to maintain cell viability in conditions of starvation or of OXPHOS dysfunction. When the bioenergetic stress lingers on, AMPK reprograms metabolism for allowing cells to tackle prolonged energy crises. Therefore, a sustained AMPK activation increases FAO as a bioenergetic source, while limiting glucose and lipid synthesis (67) and increasing mitochondrial biogenesis via PGC-1α, thus allowing a high degree of metabolic plasticity to cells undergoing energetic stress (1).

The protein complex mTORC1 is formed by the serine/threonine protein kinase mTOR, by the regulatory proteins Raptor, which facilitates substrate recruitment to mTORC1, and mLST8, which associates with the catalytic domain of mTORC1, and by the two inhibitory subunits PRAS40 and DEPTOR (86). mTORC1 promotes all major anabolic pathways, including protein, lipid and nucleotide synthesis, together with glucose metabolism and organelle biogenesis. It is activated by the Ras/ERK and by the PI3K/Akt signaling pathways, which are deregulated in most cancer types, whereas it is inhibited by the tumor suppressors p53 and LKB1. Thus, mTORC1 induction is a crucial event in the metabolic rewiring of tumor cells (66) (Figure 3). mTORC1 promotes a shift from OXPHOS to glycolysis, and it enhances the expression of many glycolytic genes by increasing HIF1α translation (87); in addition, it activates GDH, thus inducing glutaminolysis (88), and it upregulates MTHFD2 expression and therefore the mitochondrial folate pathway required for purine synthesis (89). The mTORC1 complex also activates SREBP, thus opposing the effect of AMPK on lipid metabolism and promoting lipidogenesis (90).

### Ras and mitochondrial metabolism

RAS genes encode a family of GTPases whose mutations are frequently causative of tumor development and are common in cancer. Hyperactivation of Ras-induced transduction pathways boost cell growth and proliferation, prompt resistance to death signals and promote the acquisition of invasive and metastatic properties (91, 92). Moreover, deregulated Ras signaling orchestrates pro-neoplastic changes in several components of the tumor microenvironment, including cancer-associated fibroblasts, endothelial, inflammatory and immune cells (93). Therefore, Ras mutations are among the most problematic oncogenic events, frequently associated with a dismal prognosis (94). Oncogenic activation of Ras signaling has a profound impact on the metabolic changes of tumor cells (Figure 3). It induces HIF1α both by upregulating the HIF1A gene and by enhancing translation of its mRNA via activation of the mTORC1 complex through the Ras downstream effectors ERK and PI3K/Akt (91). As described above, HIF1α in turn promotes both the transport and the glycolytic utilization of glucose and the funneling of glycolytic intermediates into the PPP (95).

In parallel, oncogenic Ras signaling affects mitochondrial bioenergetics in several ways. It prompts mitochondrial translocation of phosphoglycerate kinase I, which inhibits PDC via activation of the PDC inhibitor PDK1; as a result, less pyruvate is funneled in the TCA cycle, leading to a decrease in ROS levels together with a surge in lactate extrusion (96). K-Ras-dependent transformation inhibits OXPHOS by down-regulating respiratory complex I content (97, 98). ERK, a crucial Ras effector that can locate in mitochondria (35, 99), decreases the activity of respiratory complex II, *aka* succinate dehydrogenase (SDH; see also section Post Translational Regulation In Cancer Metabolism) (100). Nonetheless, transformation by Ras does not abrogate OXPHOS activity, which still generates a large fraction of cellular ATP (101–103).

Several evidences underline the importance of mitochondrial activity in Ras-driven tumor cells. Together with loss of p53, hyperactive Ras elicits autophagy in a non-small-cell lung cancer setting, thus preserving a proper mitochondrial function that is required for lipid homeostasis and tumor growth (104). Inhibition of the mitochondrial transcription factor Tfam leads to mitochondrial depletion and impedes growth of K-Ras-dependent lung tumors (105). Similarly to Myc-transformed cells, Ras-driven cancers strongly rely on glutamine for growth and survival (106, 107), and glutamine is the major carbon source for the TCA cycle when Ras is activated (95). In pancreatic cancer, which is characterized by activating mutations in K-Ras in more than 90% of cases, mitochondrial glutamate-oxaloacetate transaminase 2 (GOT2) utilizes glutamine-derived glutamate and oxaloacetate (OAA) to generate aspartate that is then exported to cytosol. Here, GOT isoform 1 converts back aspartate to OAA, which provides malate and finally pyruvate by malic enzyme (ME). This reaction produces NADPH, a key factor to reduce glutathione in order to avoid oxidative stress (108). Anchorage-independent growth of K-Ras-transformed cells requires α-KG generation by glutamine through glutaminase and alanine aminotransferase activity (105). Lung cancer cells exhibit a high channeling of glycolytic metabolites into the TCA cycle and glutathione biosynthesis, leading to protection from oxidative insults. This metabolic rewiring occurs when neoplastic cells are homozygous for K-Ras^G12D^, but not in heterozygous K-Ras^G12D^ cells, which characterize early tumor stages, thus highlighting the importance of oncogenic Ras in shaping metabolic heterogeneity and adaptions of cancer cells (109).

## Mutations of mitochondrial enzymes in cancer metabolism

**Figure 4:**
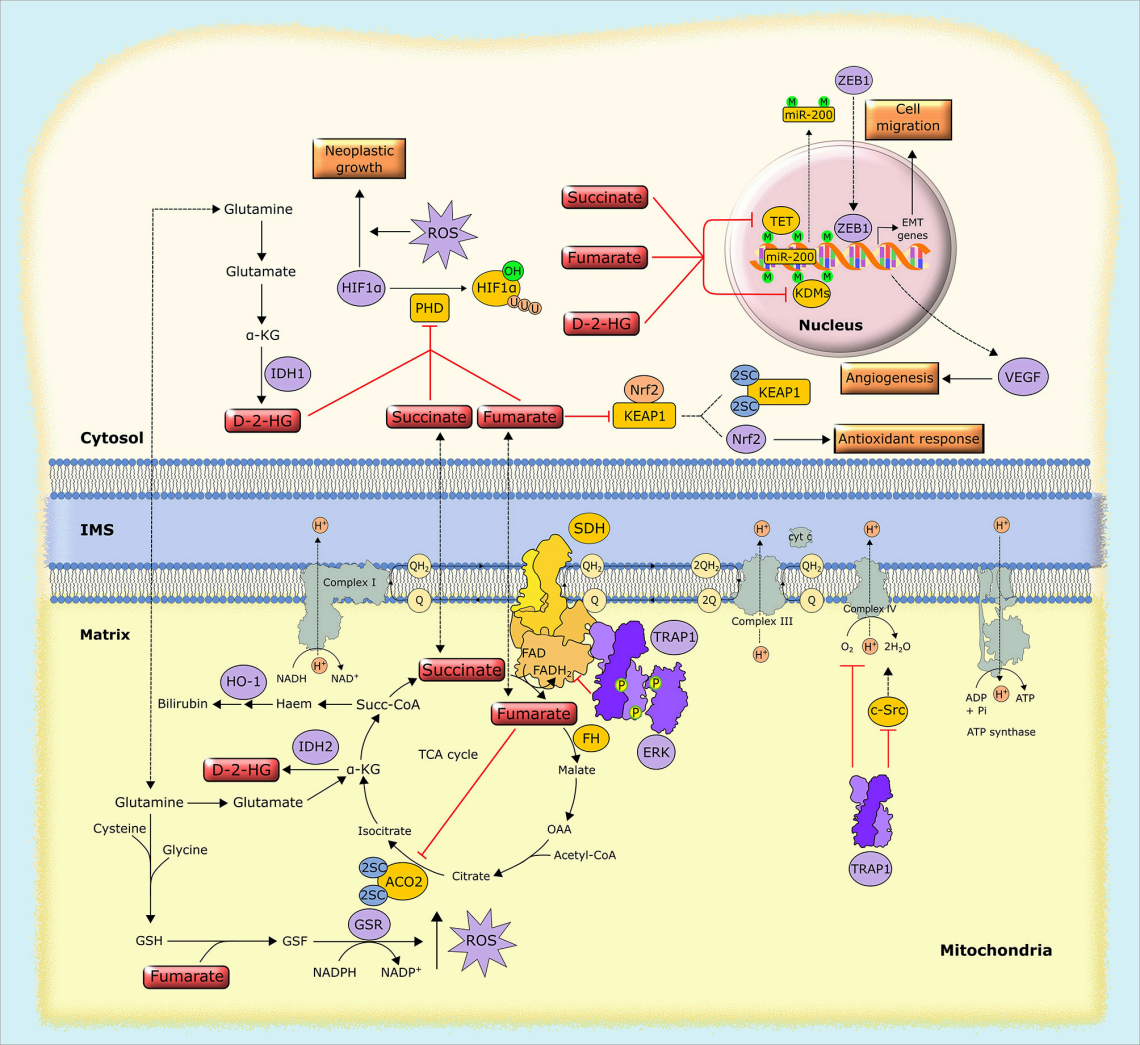
Biochemical mechanisms of oncometabolite accumulation. Inactivating mutations in genes encoding succinate dehydrogenase (SDH) and fumarate hydratase (FH), as well as oncogenic mutations in isocytrate dehydrogenase (IDH), lead to the accumulation of succinate, fumarate and D-2-hydroxyglutarate (D-2-HG). These oncometabolites inhibit α-KG-dependent dioxygenases, including prolyl hydroxylases (PHDs), the ten-eleven translocation (TET) family of methylcytosine hydroxylases and histone lysine demethylases (KDMs), leading to HIF1α stabilization and alterations in gene expression through epigenetic modifications. Separately, fumarate inactivates by succination both aconitase 2 (ACO2) and Kelch-like ECH associated protein 1 (KEAP1), which results in the activation of the antioxidant pathway mediated by NRF2, and also generates succinated glutathione (GSF), an alternative substrate to glutathione reductase (GSR). Additionally, tumor cells can use alternative ways to increase oncometabolite concentration, such as the upregulation of the mitochondrial chaperone TRAP1, which inhibits the activity of SDH, leading to the intracellular accumulation of succinate. 2SC, succination of cysteine residues; EMT, epithelial-to-mesenchymal transition; GSH, reduced glutathione; HO-1, heme oxygenase-1; IMS, intermembrane space, M, methylation; OAA, oxaloacetate; P, phosphorylation, Succ-CoA, succinyl-CoA; U, ubiquitination.

The oncogenic role played by changes in mitochondrial metabolism was first spotted by finding that mutations in subunits of SDH, an enzyme placed at the crossroad between OXPHOS and TCA cycle, as well as in the TCA cycle enzyme fumarate hydratase (FH), are causative of some human tumor types (110–113). These were paradigm-shifting findings, as they demonstrated for the first time that mitochondria, and in particular core bioenergetic enzymes, could play an active role in the complex alterations of biochemical circuitries that lead to tumor onset (114). SDH and FH act as classical tumor suppressors (Figure 4). Inactivating mutations in SDH subunits have been identified in both genetic and sporadic cancer types, including familial paraganglioma/pheochromocytoma (PGL/PCC) (115), renal carcinoma, thyroid cancer, neuroblastoma, gastrointestinal stromal tumor, ovarian cancer and testicular seminoma (116), whereas FH loss-of-function hallmarks hereditary leiomyomatosis and renal cell cancer (HLRCC) and skin and uterine leiomyomas (22).

Research in the last few years allowed understanding that the metabolic origin of this peculiar subset of neoplasms illustrates concepts of general importance. Indeed, it emerged that there is a tight intertwining between metabolism and gene expression, as cells and tissues (and neoplasms make no exception) must strictly coordinate genome expression and metabolic state for the proper unfolding of their biological routines (117). Components of intermediary metabolism affect the activity of chromatin-modifying enzyme (Figure 4). This leads to epigenomic changes mastering pro-neoplastic molecular rewiring via gene expression regulation (118). Inactivating mutations of SDH and FH drive such mechanisms by causing accumulation of their substrates succinate and fumarate, respectively, which leads to inhibition of a class of enzymes called α-KG–dependent dioxygenases. To hydroxylate their substrates, these enzymes take up one oxygen atom by α-KG, which therefore acts as a co-substrate that is decarboxylated and releases carbon dioxide and succinate. Both succinate and fumarate competitively inhibit α-KG–dependent dioxygenases, including the JmjC domain-containing demethylases (KDMs), which hydroxylate lysine residue on histones, the TET (ten-eleven translocation) family of 5-methylcytosine hydroxylases, which induce DNA demethylation of CpG islands near gene promoters, and prolyl hydroxylases (PHDs), which prompt proteasomal degradation of HIF1α (119). These enzymes play central roles in epigenetic and transcriptional regulation, and their inhibition by high levels of succinate and fumarate suppresses differentiation and promotes proliferation and further metabolic changes (20), thus inducing tumorigenesis. As a consequences of these pro-neoplastic effects, both succinate and fumarate have been dubbed oncometabolites (23, 24) and similar patterns of epigenomic changes can be observed both in FH- and in SDH-deficient tumors (120). Oncometabolites also favor tumor cell motility by affecting extracellular matrix composition via inhibition of collagen prolyl-4-hydroxylases (119) and elicit angiogenesis through succinate-dependent transcriptional induction of VEGF (116).

Fumarate inactivates aconitase 2 (Figure 4), a TCA cycle enzyme containing an iron-sulfur group (121) and proteins involved in the biogenesis of iron-sulfur clusters (122), and it inhibits the enzymatic activity of SDH through a product inhibition effect, leading to down-modulation of both OXPHOS and TCA cycle function. In order to cope with the absence of a functional TCA cycle, FH-deficient cells utilize fumarate in a linear pathway starting with glutamine and ending with the biosynthesis and degradation of haem and eventually with bilirubin excretion. This pathway is crucial for mitochondrial NADH production, and makes FH-deficient cells dependent on heme oxygenase activity, thus creating a metabolic vulnerability (123). In addition, TET inhibition by fumarate causes inactivating hypermethylation of the anti-metastatic miRNA cluster miR-200, thus promoting the transcriptional program mastered by the miR-200 target ZEB1. ZEB1 induces epithelial-to-mesenchymal transition (EMT), a complex biological rearrangement by which tumor cells acquire invasive properties (124). Thus, fumarate-dependent inhibition of miR-200 boosts EMT (125) (Figure 4). Fumarate can also tune cell redox equilibrium via protein succination, *i.e*., the interaction between fumarate and cysteine residues to produce S-(2-succino)-cysteine, which hampers protein function (24). Fumarate both inactivates by succination Kelch-like ECH-associated protein 1 (KEAP1), which promotes proteasomal degradation of NRF2, the master transcriptional regulator of the cell response to oxidative stress (23, 126), and generates succinated glutathione, an alternative substrate to glutathione reductase that leads to an increase in mitochondrial ROS levels and to HIF-1 activation (127) (Figure 4).

More recently a third oncometabolite was identified, D-2-hydroxyglutarate (D-2-HG). D-2-HG accumulates to millimolar concentrations in tumors with monoallelic mutations in IDH1 and IDH2, NADP^+^-dependent homodimers localized in cytoplasm and mitochondria, respectively, that convert isocitrate to α-KG (Figure 4). Mutant IDH1/2 dimerize with the wild-type protein and the heterodimeric enzyme acquires a neomorphic activity: the reduction of α-KG to D-2-HG in the presence of NADPH (128). As for fumarate and succinate, D-2-HG has a molecular structure that is similar to α-KG. Thus, D-2-HG acts as a competitive inhibitor of α-KG on the activity of α-KG-dependent dioxygenases (129). This results in global remodeling of DNA methylome, with an increase in methylation of both CpG islands and histones (130), and in inhibition of the PHDs that degrade the HIFs (131). Mutant versions of cytoplasmic and mitochondrial IDH isoforms are present in a fraction of acute myeloid leukemias and in the majority of glioblastomas, as well as in chondrosarcomas and cholangiocarcinomas (131). Cells harboring high levels of D-2-HG are endowed with glutamate depletion, probably because glutamate is utilized to produce α-KG and subsequently converted to D-2-HG. Both low glutamate levels and a higher NADP^+^/NADPH ratio caused by increased consumption of NADPH could suppress glutathione synthesis and regeneration, affecting the redox equilibrium of IDH mutant cells (9, 15, 24).

In addition to genetic mutations that affect the activity of enzymes involved in raising levels or generating oncometabolites, it is possible that tumor cells utilize more subtle ways to rapidly tune oncometabolite concentration, in order to afford rapid and flexible response to environmental changes. The molecular chaperone TRAP1 provides such an example. TRAP1 is a component of the HSP90 chaperone family whose expression is restricted to mitochondria and is increased in many malignancies (132). TRAP1 down-regulates the activity of both SDH (133) and respiratory complex IV (134), thus decreasing oxygen-coupled ATP synthesis and shifting the burden of ATP production to glycolysis (Figure 4). Inhibition of SDH by TRAP1 leads to succinate accumulation (133). Such an inhibition is further enhanced when ERK phosphorylates TRAP1, as in cells lacking the Ras GTPase-activating protein neurofibromin (100) that are characterized by deregulated induction of the Ras/ERK1/2 signaling pathway and form tumors in patients with the genetic syndrome neurofibromatosis type I (135). An increase in succinate levels induced by TRAP1 drives HIF1α stabilization independently of oxygen levels (136), *i.e*., it generates conditions of pseudohypoxia, an adaptive feature of many tumors that allow them sustaining the neoplastic process even before hypoxic conditions are encountered by the growing malignancy. The importance of providing such a metabolic adaptableness is highlighted by the observation that abrogating TRAP1 expression ablates tumorigenicity when different cell types are xenografted in mice (100, 133), even though the specific importance of TRAP1 in neoplastic growth could be context-dependent (132).

## Redox homeostasis and mitochondrial metabolism in tumors

**Figure 5:**
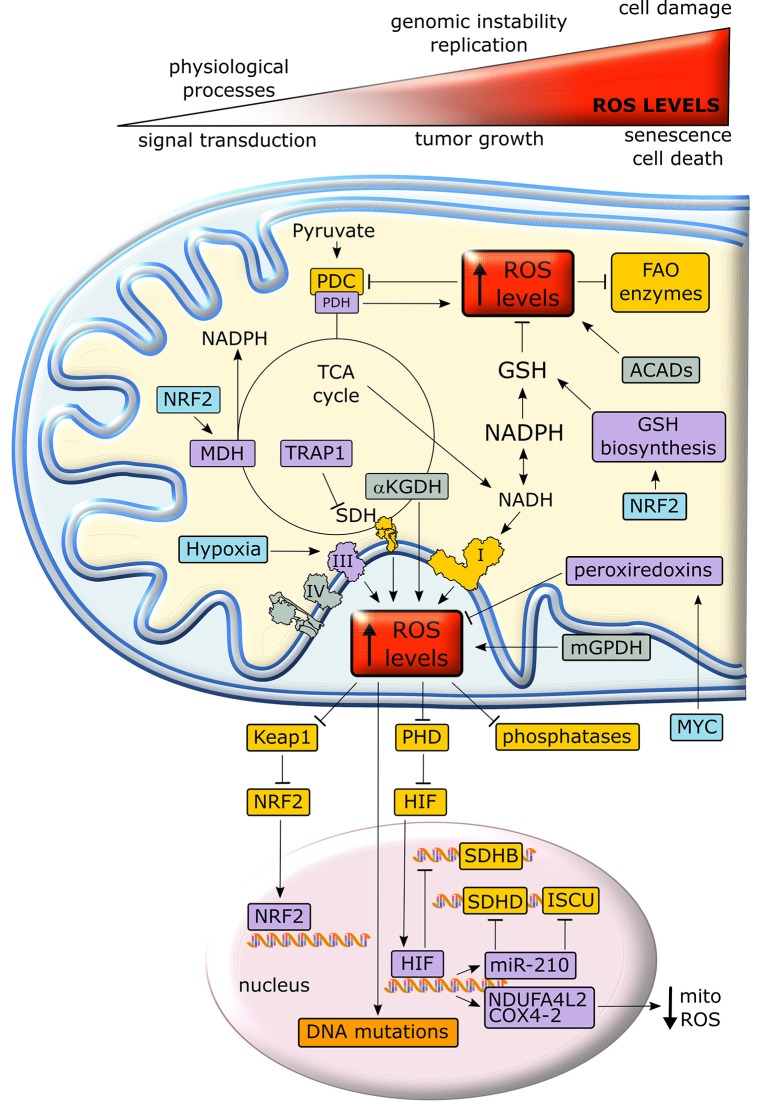
Crosstalk between metabolism and redox homeostasis in cancer cell mitochondria. Neoplastic cells are characterized by high levels of mitochondrial ROS. Under a certain threshold, ROS facilitate tumor growth, but their excessive rise elicits oxidative damage and cell death. In cancer cell mitochondria, ROS levels are increased by respiratory chain complexes, αKGDH, mGPDH and ACADs and inhibit key enzymes of lipid metabolism and TCA cycle. In turn, ROS stimulate antioxidant defenses through stabilization of the transcription factors NRF2 and HIF1α. Proteins overexpressed or activated in cancer cells are indicated in purple, whereas proteins whose activity is down-regulated are shown in yellow. GSH, reduced glutathione; PDC, pyruvate dehydrogenase complex; PDH, pyruvate dehydrogenase; αKGDH, alpha-ketoglutarate dehydrogenase; SDH, succinate dehydrogenase; SDHB, succinate dehydrogenase subunit B; SDHD, succinate dehydrogenase subunit D; MDH, malate dehydrogenase; TRAP1, TNF receptor-associated protein 1; FAO, fatty acid oxidation; ACAD, acyl-CoA dehydrogenase; mGPDH, mitochondrial glycerol-3-phosphate dehydrogenase; NRF2, nuclear factor-E2-related factor 2; PHD prolyl hydroxilase; Keap1, Kelch-like ECH-associated protein 1; NDUFA4L2, NADH dehydrogenase 1 alpha subcomplex, 4-like 2; COX4-2, Cytochrome c OXidase subunit 4 isoform 2; ISCU, Iron–Sulfur cluster assembly proteins.

Mitochondria are the major source of intracellular reactive oxygen species (ROS), as about 1% of O_2_ consumed by OXPHOS undergoes a one-electron reduction that forms a superoxide anion (137, 138). In addition, other mitochondrial metabolic enzymes, such as αKG dehydrogenase, PDH, the mitochondrial form of glycerol-3-phosphate dehydrogenase and acyl-CoA dehydrogenase can generate ROS (139–141). An excessive oxidant challenge damages biomolecules and leads to DNA mutations that eventually prompt cell senescence or death (142), but maintenance of a physiological redox equilibrium, or oxidative eustress (141), governs a variety of life processes and signal transduction pathways (137) (Figure 5).

Intracellular ROS levels are in general higher in tumor cells than in their non-transformed counterparts, and are involved in oncogene activation, tumor suppressor loss, metabolic rewiring, mutations in mitochondrial DNA (mtDNA) or hypoxia (142). ROS can reversibly target cysteine residues within the enzymatic sites of many phosphatases, such as the PI3K inhibitor PTEN, MAPK phosphatases and Tyr phosphatases (143), causing their inactivation. The consequent boost of kinase signaling pathways (144) affects mitochondrial metabolism, e.g. by tyrosine kinases that inhibit at multiple levels the PDC (145). In addition, many mitochondrial FAO enzymes contain ROS-sensitive Cys residues (Figure 5). Taken together these observations suggest that oxidative stress can tune mitochondrial metabolism by compromising both beta-oxidation of lipids and pyruvate entry into the TCA cycle (139). mtDNA mutations deregulate redox equilibrium by hampering respiration. Such mutations prompt *in vitro* and *in vivo* tumorigenicity, correlate with acquisition of metastatic potential and poor prognosis and can be used for cancer detection and determination of the degree of malignancy (20).

Hypoxia increases superoxide release from respiratory complex III, leading to PHD inhibition, possibly via oxidation of Fe^2+^ that is required for PHD function, and to the ensuing stabilization of HIFα subunits (52). In turn, HIF activation decreases ROS production by (i) down-modulating OXPHOS activity, as it suppresses SDHB expression (146), induces NDUFA4L2 (NADH dehydrogenase 1 subcomplex, 4-like 2), which attenuates complex I activity (147), and prompts the substitution of the COX subunit 4-1 with COX4-2, hence optimizing COX activity in low oxygen conditions (148); (ii) up-regulating miR-210, which orchestrates inhibition of mitochondrial bioenergetics by targeting the SDHD transcript and by repressing the iron–sulfur cluster assembly proteins that are required for the incorporation of [4Fe-4S] and [2Fe-2S] groups in respiratory complexes I, II and III; (iii) down-regulating mitochondrial biogenesis via c-Myc inhibition (147); (iv) inducing mitophagy through BNIP3, Bcl-2 and BN67IP3L/NIX induction (149). In keeping with this last point, absence of the mitophagy inducer Parkin enhances ROS generation by the persistence of dysfunctional mitochondria and increases tumorigenesis in multiple cancer models (150).

If activation of mitochondrial ROS generation remains below what triggers manifest cellular damage, it can contribute to the neoplastic process by causing DNA damage and genomic instability or by prompting dysregulated activation of crucial signaling pathways, eventually impacting on cell proliferation, angiogenesis and invasiveness (143). As an example, anchorage-independent growth of K-Ras-transformed cells requires an increase in mitochondrial ROS generated by respiratory complex III (105). Therefore, neoplastic cells must enhance their antioxidant devices, such as the tripeptide glutathione (l-glutamyl-l-cysteinyl-glycine), in order not to reach a threshold of oxidative damage incompatible with their survival. Both glutamine-derived glutamate and glucose-derived glycine are substrates for glutathione biosynthesis. NADPH, which is essential for the regeneration of reduced glutathione, is similarly obtained either by glucose through PPP and serine metabolism, or by glutamine via ME. Other anti-oxidant systems such as peroxiredoxins, which are induced by MYC, are also highly expressed in many cancer types (139).

The transcription factor Nrf2 (nuclear factor-E2-related factor 2) is a master regulator of cell response to oxidants that undergoes proteasomal degradation following ubiquitination by KEAP1. Under oxidative stress KEAP1 is inactivated, thus allowing nuclear accumulation and activation of Nrf2 (141) (Figure 5). Nrf2 induces enzymes that enhance carbon flux from glutamine toward GSH biosynthesis, utilization and regeneration, and stimulates NADPH production, e.g., by controlling ME, which boosts the oxidative decarboxylation of malate to pyruvate in order to feed the TCA cycle (142). Activation of Nrf2 increases mitochondrial membrane potential, FAO, ATP levels, rate of respiration and efficiency of oxidative phosphorylation (151). These protective functions against oxidative insults suggest that Nrf2 acts as a tumor suppressor, and indeed Nrf2 activation is beneficial in cancer chemoprevention and Nrf2-deficient mice are more sensitive to chemical carcinogenesis; in addition, the absence of Nrf2 has been related to a high metastatic potential (152). Nonetheless, the role of Nrf2 on tumorigenesis is highly contingent, as Nrf2 knockout mice are protected against tumor formation in the stomach, bladder, and skin (153) and activation of the Nrf2/KEAP1 system by somatic mutations is associated with a poor prognosis in patients (152) and has been observed in several cancer types. For instance, induction of the Nrf2/KEAP1 pathway occurs in the very early steps of hepatocarcinogenesis in the resistant hepatocyte rat model, where it associates with a metabolic rewiring toward increased glucose utilization, PPP activation and OXPHOS inhibition (154). In this model also the mitochondrial chaperone TRAP1 is highly expressed from the initial, pre-neoplastic lesions (154), and it probably contributes to the anti-oxidant mechanisms of tumor cells by decreasing SDH-generated ROS (155). TRAP1 has an oncogenic activity and its expression is induced in a variety of tumor types; however, TRAP1 levels decrease in the advanced stages of a small set of epithelial cancers (132). These contrasting observations on the role of Nrf2 and TRAP1 in neoplastic progression suggest that changes in cell redox equilibrium might have different effects on tumorigenesis, probably depending on tumor type and stage.

## Mitochondrial Ca^2+^ and metabolic plasticity

**Figure 6:**
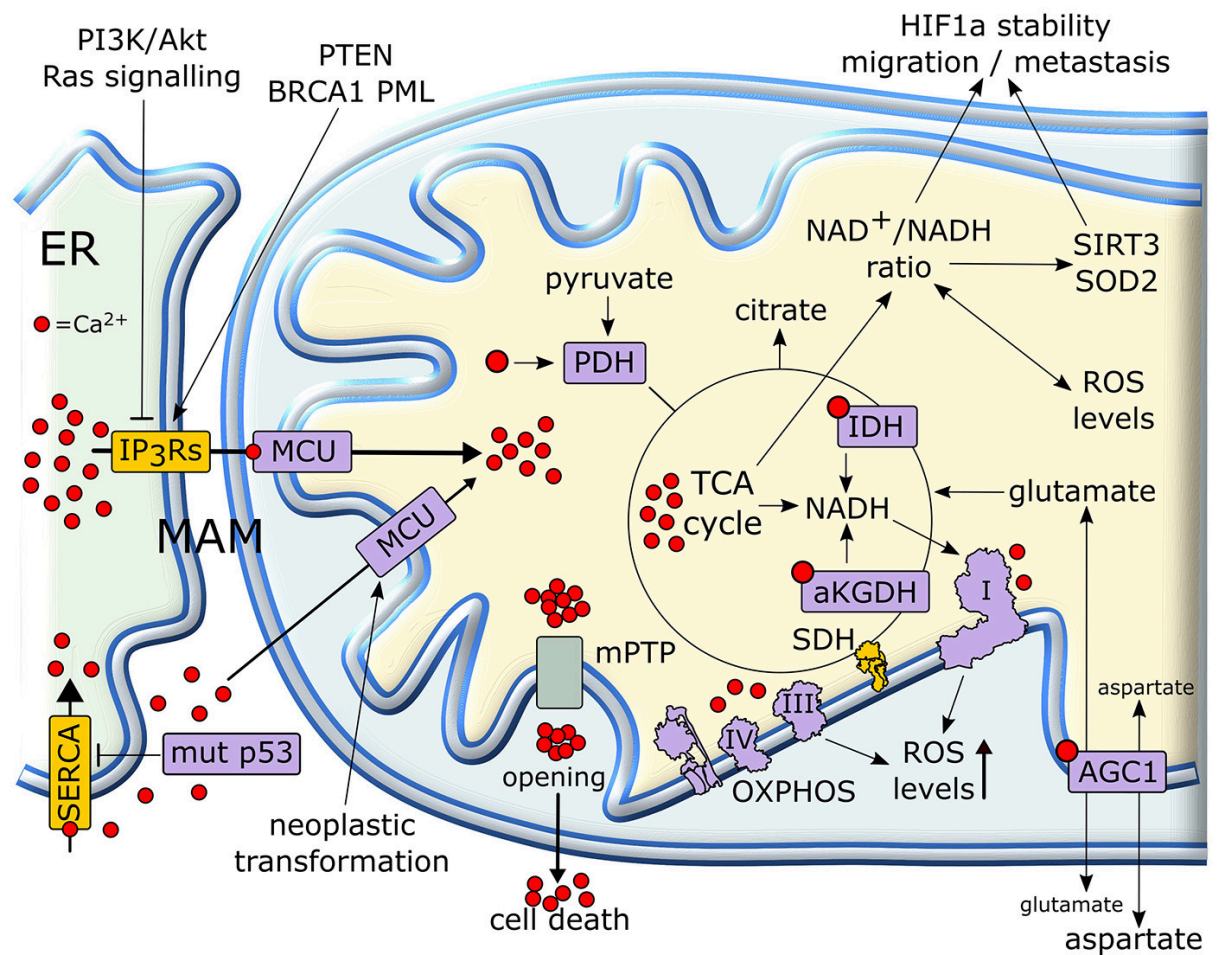
Mitochondrial Ca^2+^ in the regulation of tumor cell metabolism. Ca^2+^ released from (ER) is taken up by mitochondria, where it increases the activity of TCA cycle and OXPHOS. In cancer cells, a rise in matrix Ca^2+^ can stimulate production of metabolic intermediates, glutamate transport and NADH formation for antioxidants defenses. NADH formation can also influence the pro-neoplastic stabilization of HIF1α. Several proteins with pro- or anti-neoplastic activity regulate IP3R Ca^2+^ channels in MAMs in order to prevent matrix Ca^2+^ overload, mPTP opening and the consequent cell death. Proteins overexpressed or activated in cancer cells are indicated in purple, whereas proteins whose activity is down-regulated are shown in yellow. ER, endoplasmic reticulum; IP3R, inositol 1,4,5-triphospate receptor; MCU, mitochondrial calcium uniporter; mPTP, mitochondrial permeability transition pore; PDH, pyruvate dehydrogenase; SDH, succinate dehydrogenase; αKGDH, alpha-ketoglutarate dehydrogenase; IDH, isocitrate dehydrogenase; HIF, hypoxia-inducible factor; SERCA, sarco/endoplasmic reticulum Ca^2+^-ATPase.

Calcium ions are intracellular second messengers that tune a variety of fundamental cell processes (156). Mitochondria can accumulate high amounts of Ca^2+^, thus acting both as Ca^2+^ stores that control the spatial and temporal shape of Ca^2+^-mediated cellular signals, and as effectors that utilize Ca^2+^ to regulate cell survival, proliferation, redox state and metabolic changes. Mitochondrial Ca^2+^ homeostasis requires an efficient interplay between endoplasmic reticulum (ER), where most intracellular Ca^2+^ is stocked, and mitochondria in specialized microdomains called MAMs (Mitochondria-Associated Membranes) (157). In MAMs, Ca^2+^ is released from ER through IP3Rs (Inositol 1,4,5-triPhosphate Receptors) and is taken up by Mitochondrial Calcium Uniporter (MCU) complex, thus increasing Ca^2+^ concentration in mitochondrial matrix (158) (Figure 6).

Mitochondrial Ca^2+^ homeostasis is dysregulated in most neoplastic cells and contributes to their adaptations to stressful conditions in a fast and flexible way. Transduction pathways deregulated in cancer, such as PI3K/Akt or Ras signaling, can limit Ca^2+^ flux to mitochondria inhibiting IP3Rs, whereas tumor suppressors such as PTEN, BRCA1 or PML favor Ca^2+^ release from IP3Rs and the subsequent increase in mitochondrial Ca^2+^ levels (159, 160). Oncogenic mutations in p53 decrease the activity of SERCA (sarco/endoplasmic reticulum Ca^2+^-ATPase), which takes Ca^2+^ up in ER, thus enhancing Ca^2+^ transfer to mitochondria in MAMs (160). Rapid spikes of Ca^2+^ levels in mitochondrial matrix induce the permeability transition pore (PTP), a mega-channel formed by ATP synthase whose prolonged opening elicits a sudden cell death (161). Thus, by slowing-down mitochondrial Ca^2+^ entry through MAMs, tumor cells can avoid to succumb to several noxious stimuli (162, 163). Hence, a fine tuning of IP3Rs activity is crucial in preventing lethal matrix Ca^2+^ overload. A complex interplay exists between IP3R regulation and Ca^2+^ homeostasis in mitochondria in tumors. Indeed, inhibition of Ca^2+^ transfer from ER to mitochondria decreases the viability of tumor cells compromising their bioenergetics. Notably, in these conditions neoplastic cells activate autophagy as a salvage mechanism, but this turns out to be insufficient for their survival (164).

More controlled raises in matrix Ca^2+^ concentration have important metabolic effects, as Ca^2+^ enhances the activity of mitochondrial dehydrogenases of the TCA cycle, IDH and αKGDH, and of PDH (165). These dehydrogenase reactions lead to formation of NADH that carries the reducing equivalents required for OXPHOS activity (158). Therefore, mitochondrial Ca^2+^ stimulates respiration and increases ROS generation (166). Moreover, Ca^2+^ stimulates the aspartate/glutamate exchanger in the inner mitochondrial membrane (167), further boosting TCA cycle activity by increasing matrix glutamate levels. Taken together, these observations suggest that lowering mitochondrial Ca^2+^ concentration could play a key role in maintaining a “Warburg-like” phenotype in neoplastic cells, while protecting them from PTP opening. However, the few studies that have directly assessed the role of mitochondrial Ca^2+^ in the tumorigenic process sketch elements of a more complex picture. For instance, the expression of MCU, whose activity can sharply increase mitochondrial Ca^2+^ concentration (158), is unexpectedly increased and associated to poor prognosis, invasiveness and metastasis in models of breast cancer and hepatocellular carcinoma (168, 169). Indeed, these observations directly link MCU activity to the maintenance of redox homeostasis. MCU silencing in triple negative breast cancer models decreases ROS levels by lowering ATP production and NADH cellular content. This hampers HIF-1α stability and transcriptional activity, decreasing cell motility and invasiveness, tumor growth, lymph node infiltration and lung metastasis (169). In HCC cells, a MCU-dependent increase in matrix Ca^2+^ concentration stimulates TCA cycle activity and augments NADH/NAD^+^ ratio. This inhibits a Sirtuin3/superoxide dismutase 2 axis that boosts mitochondrial ROS levels, which in turn sustain invasion and metastasis of hepatocellular carcinoma cells in an *in vivo* xenograft model (168).

Further studies are clearly needed to dissect how frequencies and amplitudes of mitochondrial Ca^2+^ oscillations influence the metabolic changes that characterize tumorigenesis.

## Post translational regulation in cancer metabolism

Mitochondria can utilize post-translational modifications (PTMs) of their proteins in order to harmonize their activity to environmental conditions. A wide assortment of PTMs can lead to conformational changes in the tertiary structure of mitochondrial proteins, tuning their activity in response to changes in nutrient availability or redox conditions (170, 171) and furnishing cancer cells with a broad array of accurate and rapid metabolic adaptations (171). The investigation of these regulatory networks, and the functional connection with the metabolic changes that characterize neoplastic cells is complex and still in its infancy, and we will provide here only some general information.

The most prevalent mitochondrial PTM is acetylation of lysine residues, presumably because it requires acetyl-CoA that is highly compartmentalized in mitochondria (172). About 30% of mitochondrial proteins can undergo reversible acetylation. In general, this is an inhibitory mark for metabolic enzymes, as it would serve to sense the overproduction of acetyl-CoA, thus providing a negative feedback to mitochondrial metabolic circuitries that operate in an oxidative mode (173). Notably, hyperacetylation of mitochondrial proteins is observed in many diseases, including cancer (170). Acetylation is determined by the balance between the activity of acetyltransferases and deacetylases. Little is known on mitochondrial acetyltransferases. The only candidate is GCN5L1, which does not contain an acetyltransferase catalytic domain but promotes protein acetylation in the presence of acetyl-CoA, and its genetic disruption down-regulates acetylation of mitochondrial proteins. GCN5L1 is involved in lipid metabolism, as its induction promotes FAO, even if no data on tumor models are at present available (174).

Deacetylation is carried out by a class of enzymes called sirtuins, a protein family composed by 7 members, three of which (SIRT3–5) have a mitochondrial localization. Recent work has demonstrated that sirtuins are indeed deacylases, as they are able to transfer a variety of long acyl moieties including succinyl, malonyl, ADP-ribosyl and lipoyl groups, in addition to perform deacetylase reactions. All these reactions require NAD^+^, thus linking sirtuin enzymatic activity to the metabolic state of the cell and poising them as metabolic stress sensors (175). Mitochondrial sirtuins orchestrate the coordinated regulation of substrate clusters, in order to efficiently tackle conditions of metabolic stress. SIRT3 is the major mitochondrial deacetylase and is activated upon starvation and by increased NAD^+^ levels. In these conditions, SIRT3 enhances oxidative metabolism of fatty acids, by activating LCAD, and of amino acids, by increasing the activity of GDH and GLS2 (176, 177). SIRT3 also activates the PDH complex (PDC), thus promoting the conversion of pyruvate to acetyl-CoA (178). In parallel, SIRT3 stimulates ROS-mitigating systems such as IDH2, a TCA cycle enzyme that generates NADPH required to reduce glutathione (175), and superoxide dismutase 2, which converts superoxide to hydrogen peroxide that is then neutralized by glutathione (173). In addition, SIRT3 activates by deacetylation all OXPHOS complexes, in particular complex I and SDHA, the entry point of electrons from NADH and FADH2, respectively, thus promoting an efficient respiration (175). Taken together, these observations indicate that SIRT3 opposes a Warburg-like metabolism, and cells lacking SIRT3 exhibit genomic instability and are prone to tumorigenesis in xenografts. Accordingly, Sirt3-knockout mice develop mammary tumors, and in many human cancer types SIRT3 is deleted or expressed at a very low level (178). Nonetheless, the functional connections between mitochondrial sirtuin activity and tumor growth are multifaceted and far from being fully understood. Indeed, SIRT4 seems to have a tumor-suppressor role similar to that of SIRT3, as SIRT4-null mice develop lung tumors, loss of SIRT4 accelerates tumor progression in a mouse Burkitt lymphoma model and SIRT4 expression is reduced in several types of human cancers. However, SIRT4 plays opposing roles to SIRT3 in the regulation of several metabolic pathways: it promotes lipogenesis and represses fatty acid oxidation by inhibiting malonyl-CoA decarboxylase (170), negatively regulates PDC and represses GDH (177, 178). SIRT5, the last mitochondrial sirtuin, primarily demalonylates and desuccinylates lysine residues in a NAD^+^-dependent way. Its functions in the metabolic rewiring of tumor cells are poorly understood, but it might be involved in glutamine metabolism as it inhibits GLS (177) and in OXPHOS and TCA regulation, as it decreases SDH activity by targeting both SDHA and SDHB and it inhibits PDC (170). Notably, SIRT5 could act as an oncogene, as it is overexpressed and associated with poor prognosis in human lung cancer (178). Finally, a further layer of metabolic regulation could be provided by sirtuin-directed PTMs such as phosphorylations (176).

Reversible phosphorylation at serine, threonine or tyrosine residues is emerging as an important mechanism regulating several aspects of mitochondrial metabolism. For instance, the inhibitory phosphorylation of PDC via enhanced expression of pyruvate dehydrogenase kinase-1 contributes to aerobic glycolysis and malignant phenotype, whereas PDH phosphatase exerts the opposite effect (145).

Adaptations to changes in nutrients and oxygen supply require a rapid OXPHOS regulation that can be achieved via reversible phosphorylations. The mitochondrial fraction of protein kinase A (PKA) activates in response to CO_2_ generated in the TCA cycle. PKA increases the activity of respiratory complex I through phosphorylation of its NDUFS4 subunit (179). PKA also phosphorylates a subunit of cytochrome oxidase, preventing its allosteric inhibition by ATP and acting as a metabolic sensor to match OXPHOS activity with substrate availability and energy consumption requirements (180). Several phosphorylation sites are present on ATP synthase, but how they modulate the enzyme is still largely obscure. Preliminary data indicate that they could affect not only its activity, but also its assembly and dimerization (181). Activation of the tyrosine kinase Src increases the enzymatic activity of respiratory complex IV in isolated rat brain mitochondria (182); accordingly, the Src inhibitor dasatinib down-regulates the activity of respiratory complex IV in some tumor cell models, inhibiting their ROS-dependent invasiveness (134).

Tyrosine phosphorylation of SDH subunit A by the Src-like tyrosine kinase Fgr increases SDH activity, contributing to the capacity of mitochondria to modulate metabolism in conditions of nutrient restriction or hypoxia (183). Conversely, the kinase ERK1/2 decreases SDH activity. ERK1/2, SDH and the chaperone TRAP1 form a multimeric complex in mitochondria of neurofibromin-deficient cells. Mitochondrial ERK1/2 phosphorylates TRAP1, thus enhancing its inhibition of SDH. TRAP1 ablation or mutagenesis at the Ser residues targeted by ERK1/2 abrogates the tumorigenicity of cells lacking neurofibromin (100).

The mitochondrial fraction of the Ser/Thr kinase GSK-3 down-modulates the activity of PDH and of respiratory complex I (184). Moreover, GSK-3 phosphorylates the mitochondrial chaperone cyclophilin D (CyP-D), the best characterized proteinaceous regulator of the PTP, enhancing CyP-D-dependent PTP induction. In tumor cells, mitochondrial ERK1/2 inhibits by phosphorylation GSK-3, thus antagonizing PTP opening and cell death (99). A complex array of PTMs, including acetylations and nitrosylations in addition to phosphorylation, affects CyP-D activity (162). These PTMs could subtly tune the bioenergetic status of neoplastic cells, as CyP-D binds and down-regulates the enzymatic activity of ATP synthase (161).

## Mitochondrial dynamics and cancer metabolism

Mitochondria are extremely dynamic organelles, undergoing the opposite processes of fusion and fission in a coordinated and balanced way. A comprehension of how mitochondrial dynamics contribute to the metabolic rewiring of cancer cells is still in its infancy, but several evidences are emerging that link decreased fusion and enhanced fission to neoplastic transformation, invasion and metastasis (185, 186). Signaling via oncogenic MAPK and PI3K promotes fission (187), and in certain tumor settings high levels of the fission protein DRP1, whose activity is increased by ERK-dependent phosphorylation, negatively correlate with survival of patients. Conversely, overexpression of the fusion proteins mitofusins decreases tumor growth, and their levels are directly related to OXPHOS activity and ATP production in several cell models (188).

However, the assumption that glycolytic cells have fragmented mitochondria, whereas OXPHOS is increased in cells with elongated mitochondria, appears as an oversimplification (188). Indeed, a prolonged DRP1 downregulation can inhibit respiration, suggesting that a proper OXPHOS modulation requires a balanced interplay between mitochondrial fission and fusion (189). Thus, it is difficult at present to make mechanistic correlations between mitochondrial dynamics and metabolism and to understand whether changes in the mitochondrial network of cancer cells are priming events or consequences of their metabolic rewiring. For instance, even if activation of several oncogenes increases fission, some others, such as Myc, promote mitochondrial fusion (187). Moreover, mitochondrial shape can impact on intramitochondrial Ca^2+^ waves (185) and on Ca^2+^ fluxes at MAMs (189), thus playing a complex and probably context-dependent role on the metabolic adaptations of tumor cells (see section Mitochondrial Ca^2+^ And Metabolic Plasticity).

Mitochondrial fission is strictly connected to mitophagy, a quality control process that maintains mitochondrial integrity and function through removal of damaged organelles, which must be isolated from the healthy network via DRP1-dependent sequestration (189, 190). Mitophagy is activated by a variety of stresses usually encountered by neoplastic cells, including hypoxia, nutrient deprivation, DNA damage and inflammation, which eventually cause mitochondrial membrane depolarization and decline in respiratory capability (149). Therefore, any impairment in the mitophagy process leads to accumulation of dysfunctional mitochondria, hence decreasing respiration and ATP production and increasing ROS levels. In general, defects in mitophagy affect the metabolic plasticity of mitochondria in response to environmental stresses such as altered Ca^2+^ signaling, ROS generation and changes in nutrient availability, further amplifying their noxious effects on the cell. In cancer, a disruption of the homeostatic equilibrium between mitophagy and mitogenesis occurs (191, 192). It has been proposed that impairment of a correct mitophagy could be advantageous for the early phases of neoplastic growth, contributing to set a novel redox equilibrium, whereas later stages of tumor progression would be favored by mitophagy, as it would protect tumor cells from excessive mitochondrial damage, surge in ROS levels and apoptosis (189). Further work is certainly needed to draw a more complete picture of the functional interplay between mitochondrial fusion and fission, mitophagy and metabolic rewiring of cancer cells.

## Concluding remarks

Metabolism is a multilevel process, encompassing and integrating a myriad of factors both at the organismal scale, such as age or lifestyle, and at the local level, including cellular composition of the microenvironment, nutrient supply and stiffness of the extracellular matrix. Accordingly, aberrant metabolic reprogramming in cancer is both the cause and the effect of alterations at multiple levels that reverberate on each other.

The (epi)genomic landscape of neoplastic cells, the intertwined molecular signaling between immune, stromal and other non-transformed cells with the malignant ones in tumor microenvironment (193, 194), as well as increases in hydrostatic forces and in the stiffness of the tumor milieu (195), are all factors that constantly tune cancer cell metabolism to fluctuating environmental conditions, leading to metabolic heterogeneity also across different areas of the same tumor. In neoplastic cells, mitochondria constitute a point of integration for many of these metabolic circuitries. For instance, reciprocal feedbacks exist between mechanosignaling, the process by which cells convert extracellular mechanical forces into biochemical outputs, and glutamine metabolism, as glutamine partly controls focal adhesions and actin stress fiber assembly, and in turn stiffness changes glutamine fluxes (196).

Moreover, mitochondria are at the heart of mechanisms that balance a variety of intracellular metabolic circuitries. For instance, NADPH homeostasis is maintained by several mitochondrial metabolic circuitries via TCA cycle intermediates and ATP generated by OXPHOS. These pathways include one carbon metabolism, PPP, whose oxidative branch is enhanced by citrate- or ATP-dependent inhibition of late glycolytic steps (5, 11), glutamine-derived carbons diverted out of the TCA cycle to convert malate into pyruvate via ME, and IDH (6, 197, 198). p53 represses the transcription of ME genes, thus inhibiting the usage of TCA cycle intermediates for NADPH production (68). Conversely, citrate inhibits PFK, pyruvate kinase and PDH, blocking pyruvate generation from glycolysis and leading to an increase in ME activity to maintain pyruvate levels, but also to provide NADPH (11). Another important source of NADPH is lipid β-oxidation, which becomes a major fuel for ATP synthesis during invasion and metastasis (199), further highlighting the tight connection between metabolic adaptations and biological conditions in cancer cells.

Citrate and acetyl-CoA provide other examples of multiple metabolic intersections. Mitochondrial citrate is a TCA cycle metabolite that originates by the condensation of OAA and acetyl-CoA, but it also forms via glutamine-fueled reductive carboxylation (47, 200), which is particularly important under hypoxic conditions and supports anchorage-independent growth of neoplastic cells by mitigating oxidative stress through a coordinated regulation of NADH/NADPH dependent IDH1 and 2 in cytosol and mitochondria (201). In mitochondria, citrate down-regulates SDH (11), *i.e*., the point of integration between TCA cycle and OXPHOS, contributing to raise the levels of the oncometabolite succinate. Alternatively, citrate can move to cytosol, where it both favors glucose usage in PPP and serine synthesis by inhibiting the late glycolytic steps and is converted into acetyl-CoA (11). In turn, acetyl-CoA starts lipid synthesis and sustains acetylation reactions (see section Post Translational Regulation In Cancer Metabolism) and influences a variety of biochemical circuitries involved in the neoplastic process, including histone acetylation and chromatin remodeling as well as redox homeostasis via acetylation of superoxide dismutase and IDH (1). Regulation of histone acetylation showcases the fundamental role played by metabolic enzymes and metabolites in gene expression control (117). One interesting example is provided by the PDC, which locates both in mitochondria and nucleus of prostate cancer cells. This compartmentalization is instrumental to orchestrate lipid biosynthesis both by providing cytosolic citrate and by inducing the transcription of genes for lipid synthesis by regulating histone acetylation (202).

As described in section Mutations Of Mitochondrial Enzymes In Cancer Metabolism, tumors with mutations in enzymes of the TCA cycle constitute an excellent model to study how mitochondrial metabolism causes pro-neoplastic (epi)genomic changes (203), and they also provide clues to identify molecular vulnerabilities that can be exploited for anti-tumor strategies. For instance, pyruvate carboxylase (PC) enables aspartate synthesis in SDH-deficient tumor cells, creating a metabolic vulnerability. lack of SDH activity commits cells to consume extracellular pyruvate, which sustains Warburg-like bioenergetic features. Pyruvate carboxylation diverts glucose-derived carbons into aspartate biosynthesis, thus sustaining cell growth (204, 205).

Further layers of complexity are provided by the heterogeneity of cellular components of the tumor mass. For instance, cancer stem cells (CSC) constitute a small population of self-renewal neoplastic cells that are *per se* capable of promoting tumor growth. The metabolic features of CSC differ from those of the bulky neoplasm, and recent evidences point toward an OXPHOS phenotype in CSC. In a sort of reverse Warburg metabolism, OXPHOS CSC might be fed by glycolytic tumor cells or by cells of the tumor microenvironment, such as cancer associated fibroblasts, and could switch to a glycolytic metabolism under hypoxia (206). Examples of symbiotic nutrient sharing between neoplastic cells and tumor microenvironment are observed in metastatic ovarian cancer cells, breast cancer cells or leukemic stem cells, which oxidize fatty acids supplied from surrounding adipocytes to sustain proliferation, survival and invasiveness and possibly to preserve cell redox balance (207, 208). Moreover, horizontal transfer of mitochondria from stromal to cancer cells can lead to an increase in OXPHOS metabolism of the latter (19, 209).

Metabolic plasticity of tumor cell mitochondria offers a high window of opportunity for efficient anti-cancer therapy, since transformed cells have metabolic needs that differ from their non-transformed counterparts, and molecules such PDH, IDH1/2 or glutaminase inhibitors are already in clinical trials. Nonetheless, such a plasticity can also be a hurdle when trying to develop selective therapeutic strategies. As an example, ovarian cancer cells gain resistance to antiangiogenic therapy by shifting their metabolic phenotype toward a highly glycolytic one (210). Importantly, tumor cell dependency on specific bioenergetic features *in vitro* can be extremely different from the *in vivo* situation, due to off-target effects, suboptimal pharmacokinetic properties of the compound or metabolic heterogeneity of the neoplastic mass.

A profound comprehension of the biochemical mechanisms that govern the bioenergetic flexibility of tumor cell mitochondria and its interplay with a multitude of extra-mitochondrial signals constitutes a central dowel to build an integrate model of the metabolic features that hallmark cancer. Incorporation of data obtained at different scales of analysis, from the organism to the organelle, remains a tremendous task. Nonetheless, huge advances have been recently made unveiling biochemical processes and therapeutic opportunities that were unimaginable even few years ago.

## Author contributions

All authors contributed to writing the text and drawing the figures and approved the manuscript for publication.

### Conflict of interest statement

The authors declare that the research was conducted in the absence of any commercial or financial relationships that could be construed as a potential conflict of interest.
